# Generation of High-Value Genomic Resource in Rice: A “Subgenomic Library” of Low-Light Tolerant Rice Cultivar Swarnaprabha

**DOI:** 10.3390/biology12030428

**Published:** 2023-03-10

**Authors:** Sovanlal Sahu, Payal Gupta, Thirumalanahalli Prakash Gowtham, Kumar Shiva Yogesh, Tenkabailu Dharmanna Sanjay, Ayushi Singh, Hay Van Duong, Sharat Kumar Pradhan, Deepak Singh Bisht, Nagendra Kumar Singh, Mirza J. Baig, Rhitu Rai, Prasanta K. Dash

**Affiliations:** 1 ICAR-National Institute for Plant Biotechnology, Pusa Campus, New Delhi 110001, India; 2 Institute of Agricultural Sciences for Southern Vietnam, Ho Chi Minh City 71007, Vietnam; 3 ICAR-National Rice Research Institute, Cuttack 753006, India; 4 Indian Council of Agriculture Research, Krishi Bhawan, New Delhi 110001, India

**Keywords:** rice, photoreceptors, phytochromes, far-red light, light-responsive genes, Gramineae

## Abstract

**Simple Summary:**

The present investigation into the “Generation of high-value genomic resource in rice cultivar Swarnaprabha” was carried out to isolate the phytochrome photoreceptors from low-light tolerant cultivar of rice. Two contrasting cultivars of rice (IR-64 and Swarnaprabha) were grown under low-light conditions to note the morphological differences in their responses to low-light. Based on the growth of the plants, they were confirmed to be tolerant (Swarnaprabha) and susceptible (IR-64) to light intensity. To investigate the molecular basis of low-light tolerance, the tolerant cultivar was used to construct a subgenomic library. Sixty-six clones from the library were randomly screened for the presence of genomic fragments and were found to be recombinant. Further, the constructed Swarnaprabha library is a valuable genomic resource for Gramineae community users, and it can be used as an important resource for understanding the molecular basis of the variation in the response to low-light among the photosensitive cultivars of rice. All the clones of the generated library were stored long-term to isolate and characterize the phytochromes and other intrinsic yield genes involved in the shade-avoidance response (SAR) in rice.

**Abstract:**

Rice is the major staple food crop for more than 50% of the world’s total population, and its production is of immense importance for global food security. As a photophilic plant, its yield is governed by the quality and duration of light. Like all photosynthesizing plants, rice perceives the changes in the intensity of environmental light using phytochromes as photoreceptors, and it initiates a morphological response that is termed as the shade-avoidance response (SAR). Phytochromes (PHYs) are the most important photoreceptor family, and they are primarily responsible for the absorption of the red (R) and far-red (FR) spectra of light. In our endeavor, we identified the morphological differences between two contrasting cultivars of rice: IR-64 (low-light susceptible) and Swarnaprabha (low-light tolerant), and we observed the phenological differences in their growth in response to the reduced light conditions. In order to create genomic resources for low-light tolerant rice, we constructed a subgenomic library of Swarnaprabha that expedited our efforts to isolate light-responsive photoreceptors. The titer of the library was found to be 3.22 × 10^5^ cfu/mL, and the constructed library comprised clones of 4–9 kb in length. The library was found to be highly efficient as per the number of recombinant clones. The subgenomic library will serve as a genomic resource for the Gramineae community to isolate photoreceptors and other genes from rice.

## 1. Introduction

Rice is a nutritious cereal crop [1,2] and the main source of carbohydrates, with a limited amount of protein but substantial amounts of nutrients, such as zinc and niacin [3]. Although the “Green Revolution” and breakthrough research in the field of genomics have doubled the yield of rice, we still need to increase the production to meet the projected demand in the future [4]. Sunlight is indispensable for the growth and development of all photosynthesizing plants, including rice, starting from germination till the completion of their life cycles. Moreover, rice is a photophilic plant that requires bright sunshine of >600 h for productive growth and development during its entire growth cycle. The rice yield is directly linked to the light perception and signaling that translates the prevailing environmental conditions into net photosynthesis and the allocation of photosynthates [5,6]. In addition, the rice yield is photomorphogenically influenced by solar radiation, and particularly during the last 35 to 45 days of its maturity period.

Coincidentally, low-light affects different varieties of rice in different manners. Under low-light conditions, the grain numbers in the panicles are reduced in the short-duration varieties, the spikelet sterility is increased in the medium-duration varieties, and the panicle number is reduced in the long-duration varieties. The impact of low-light is moderate on the short- and medium-duration varieties, while it is severe on the long-duration varieties, resulting in spikelet infertility, fewer panicles, and a fewer number of grains per panicle [7].

**Figure 1:**
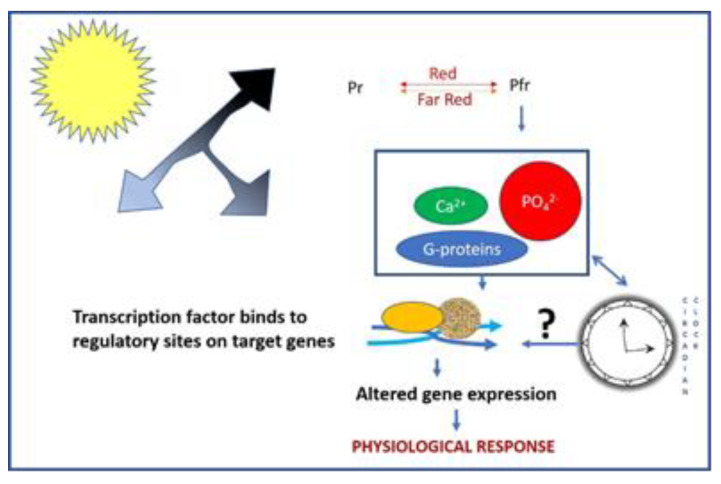
Mode of action of phytochromes in light perception in plants. The inactive Pr form in cytosol is converted to an active Pfr form by the absorption of red light, which, upon irradiation, moves to the nucleus to interact with numerous cell components to cause physiological changes in response to light. The Pfr form of phytochromes can absorb far-red light and then return to the Pr form, which implies a Pr/Pfr photo-reversibility, in which phytochromes operate as toggle switches that turn on with R light and off with FR light.

Like all plants, rice perceives light using phytochromes (PHYs), and the activated phytochromes transduce the light signals [8] to redeploy carbon resources for plant growth, architecture, flowering [9], and seed set [10]. As of today, phytochrome-modulated growth and development has been elucidated in detail in the model plant *Arabidopsis thaliana* [11] but there is limited information on cereal crop rice. Phytochromes (PHYs) are 124 kDa bili-proteins that perceive light from the environment, including the quantity, quality, and duration of the light [12]. In plants, phytochromes are biosynthesized in the physiologically inactive Pr form in the cytosol. Upon the absorption of red light, the inactive Pr form is converted to the active Pfr form and migrates into the nucleus to interact with numerous down-stream signaling components to initiate physiological changes (Figure 1) [13]. The complex phenomenology of the phytochrome function is connected to its polymorphism, the major *phy* genes being *phyA* and *phyB* [14]. Although there are five types of phytochromes (*phyA*, *phyB*, *phyC*, *phyD*, and *phyE*) in *Arabidopsis*, only three predominant types (*phyA*, *phyB*, and *phyC*) are functionally annotated in rice [15]. *PhyA* mediates irreversible photo-responses in the very-low-fluence and high-fluence ranges (VLFR and HFR), and primarily in the far-red (FR) spectral region [16], whereas *phyB* mediates the “classical” R/FR reversible responses in the low-fluence range (LFR) [17]. The *phyA* specificity is determined at three levels: (i) intramolecular events; (ii) turnover; (iii) nuclear–cytoplasmic partitioning and interaction with partner proteins [18].

**Figure 2:**
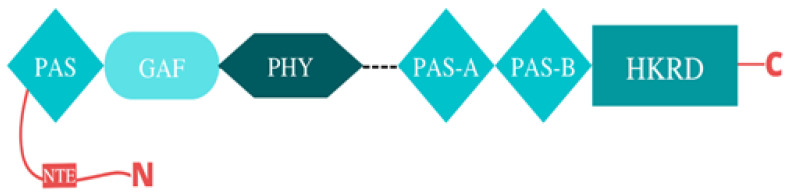
Structure of plant phytochrome. The photosensory module (PSM) of the phytochrome is predominantly made up of the conserved domains PAS, GAF, and PHY, which are responsible for photosensory activity and bilin molecule binding. The PAS and GAF domains of the photosensory core (the N-terminus) are knotted together. The PHY domain is responsible for stabilizing the Pfr chromophore. The distant end of the protein (i.e., the C-terminus), also called the output module (OPM), is made up of two domains: the PAS-A and PAS-B sub-domains (together called the PRD–PAS repeat domain), as well as the histidine kinase-related domain (HKRD), and it is responsible for dimerization. The PAS-A and PAS-B sub-domains are only found in plant phytochromes, and they form the PAS-repeat domain (PRD), whereas the HKRD domain is also found in phytochrome-like proteins.

Structurally, phytochromes are homo-dimeric modular chromoproteins with the following: (i) the C-terminal dimerization module and (ii) the N-terminal chromophore-bearing module (photosensory module (PSM)) [19,20]. The bilin-binding N-terminus is made up of three conserved domains: (1) the PAS domain; (2) the GAF domain; (3) the PHY domain. The PAS and GAF domains of the photosensory core (N-terminus) are knotted together. Molecular investigation coupled with high-resolution crystallography and in-vitro studies revealed that both the PAS and GAF domains form the photosensory core at the N-terminus and are essential for effective chromophore incorporation [21]. While both the PAS and GAF domains are implicated in other photoreceptor signaling pathways, the GAF domain is the domain that binds the bilin molecule [22]. The third and hallmark domain on the photosensory module (PSM) is the PHY domain, which is involved in stabilizing the Pfr chromophore by direct interaction with the bilin D-ring pyrrole [23] (Figure 2).

The phytochrome family of photoreceptors respond primarily to the red and far-red wavelengths, switching between Pr (inactive) and Pfr (active) isomeric forms [8,24,25,26,27,28]. The *phy* mutants in Arabidopsis exhibit reduced CO_2_ uptake and sizeable reductions in overall growth [29] and plant development. Although phytochromes have been isolated from rice, no allelic polymorphism has been studied in low-light tolerant rice genotypes. This has necessitated the creation of rich genomic resources [30], such as cDNA and gDNA libraries, allele-mining data, and genomic information, including transcriptome data.

The development of genomic resources in crop plants and plant-associated microbes have revolutionized the molecular research in the last decade, which has generated high-utility rice genomic resources in the form of the subgenomic-library-expedited isolation of desirable genes from fractionated rice genomic DNA. Because genomic libraries represent collections of clones that cover the repertoire of the genomic DNA of an organism, they are useful genetic resources for the physical mapping and cloning of agronomically important genes. Further, genomic libraries are shared as community genomic resources amongst researchers for evaluating germplasm and biological diversity [31]. In this study, we constructed a subgenomic library from a low-light tolerant cultivar of rice that can be used for the isolation and characterization of multiple light-responsive and intrinsic yield genes, including photoreceptors from rice.

## 2. Materials and Methods

### 2.1. Growth of Plants

*Oryza sativa* ssp. *indica* cultivar IR-64 and Swarnaprabha seeds procured from the NRRI, Cuttack, were sown on germination paper, and five days after germination, the seedlings were transferred to 12-inch pots containing a 1:1 mixture of cocopeat and sand, in which they continued to grow in shade and normal light at the National Phytotron Facility with a relative humidity of 75%. The light intensity was reduced by decreasing the incandescence of the LED lights to 75% of the photosynthetically available radiation (PAR: 400–700 nm) to mimic low-light conditions. The growth of both varieties was carefully noted for 18 days and 21 days after transplantation. The control rice plants of IR-64 and Swarnaprabha were raised in 12 h of light and 12 h of dark at 28 °C, with a relative humidity of 75% and 100% PAR (400–700 nm).

### 2.2. Isolation of Total Genomic DNA of Rice

The genomic DNA was extracted from the leaves of Swarnaprabha using the DNA isolation protocol, as described earlier [32]. Briefly, 0.1 g fresh leaf tissue was ground to a fine powder with the help of liquid nitrogen in a prechilled mortar and pestle. The powder was transferred to a microcentrifuge tube, followed by the addition of 500 μL of prewarmed (65 °C) CTAB extraction buffer. The sample was incubated at 65 °C for 1 h with intermittent mixing every 10 min. After cooling the sample to room temperature, an equal volume of chloroform:isoamyl alcohol (24:1) was added and mixed by inversion, followed by centrifugation at 12,000 rpm for 10 min (RT). The aqueous phase was collected in a microfuge tube, and an equal volume of chilled absolute ethanol was added to precipitate the DNA. After gentle inversion, the sample was centrifuged at 10,000 rpm for 10 min at room temperature. The supernatant was discarded, and the pellet was washed with 70% ethanol and kept for air drying. The dried pellet was then suspended in 100 μL of nuclease-free water (NFW). To remove RNA contamination from the isolated DNA, RNase A treatment was performed. The quality and quantity of the genomic DNA were checked using agarose gel electrophoresis and spectrophotometry.

### 2.3. Subgenomic Library Preparation

The subgenomic library was prepared with the aim of isolating full-length (~7 kb) phytochrome genes from Swarnaprabha. Thus, the isolated and purified genomic DNA of the rice was restricted with *Eco*RI restriction enzyme for 30–60 min at 37 °C. The restriction-digested genomic DNA was electrophoresed on a 0.8% agarose gel, and a fragment that corresponded to a range from 4 to 9 kb was excised from the gel. Partially restricted genomic DNA (4–9 kb) was eluted from the gel using a PCR purification/gel elution kit. Similarly, the pBluescript vector used for cloning the genomic fragments was prepared by restricting 2–3 μg of plasmid DNA with *Eco*RI, and it was purified from 0.8% agarose gel using a PCR purification/ agarose gel elution kit (M/s Marcherey-Nagel, Germany).

The purified *Eco*RI-restricted subgenomic rice DNA (4–9 kb) was ligated with the linearized 3 kb pBluescript vector DNA using T4 DNA ligase. The ligation mixture was incubated at 16 °C overnight. The details of the restriction of the vector DNA and ligation are presented in Table 1.

### 2.4. Screening of Recombinant Clones by α-Complementation

The recombinant vectors ligated with the insert DNA (subgenomic fragments) were transformed into chemically competent cells of the *E. coli* strain DH5α. The recombinant clones were selected on LA + carbenicillin (100 μg/mL) antibiotic plates. X-Gal (20 mg/mL) and IPTG (0.1 M) were added to the LA medium for blue–white screening. Putative recombinant clones (white colonies) were further purified on LA + carbenicillin (100 μg/mL) plates by two repeats of subculturing to maintain the library of subgenomic axenic clones of rice.

### 2.5. Estimation of Efficiency of Library by Colony PCR

The efficiency of the constructed subgenomic library was estimated by the presence or absence of the insert DNA of axenic clones of rice in it. The presence of insert DNA was confirmed by colony PCR, as described earlier [33]. The colony PCR was performed using vector-specific universal M13 forward and reverse primers (Table 2) flanking the multiple cloning site. Individual colonies were picked with the help of a toothpick from the overnight-grown colonies and were mixed in 50 μL sterile water. The colonies were heated to 95 °C for 5 min before setting up the colony PCR. The PCR components were as mentioned in Table 1, and the conditions were as follows: initial denaturation at 95 °C for 1 min, followed by 30 cycles of denaturation at 95 °C for 30 s, annealing at 60 °C for 30 s, and extension at 72 °C for 30 s.All the PCR products were visualized on 1.2% agarose gel. For the gel electrophoresis, 1.2% agarose gel containing 0.12 μg/mL ethidium bromide was prepared in 1× TAE buffer and electrophoresed at 5 V/cm for ~30 min.

The efficiency of the constructed subgenomic library was further estimated by screening for the presence of the genes of interest in the recombinant clones in the constructed subgenomic library using gene-specific primers. The presence of the genes of interest was confirmed by performing colony PCR, as described earlier [33], with a minor modification using gene-specific *phyA* (*phytochromeA*) forward and reverse primers (Table 3). Individual colonies were picked with the help of a toothpick from the overnight-grown colonies and were mixed in 50 μL sterile water. The colony was heated to 95 °C for 5 min before setting up the colony PCR. The PCR components as mentioned in Table 1 were added, and the PCR conditions were as follows: initial denaturation at 95 °C for 1 min, followed by 30 cycles of denaturation at 95 °C for 30 s, annealing at 60 °C for 30 s, and extension at 72 °C for 1 min. All PCR products were visualized on 1.2% agarose gel.

### 2.6. Long-Term Storage of Subgenomic Clones

A total of 3600 white colonies representing the subgenomic library of rice were individually picked and inoculated on 100 μL semiskirted ‘U’ plates containing 50 μL of LB + carbenicillin. The plates were incubated at 37 °C with 150 rpm for 16 h. A total of 50 μL of sterile glycerol (40%) was added to the culture, and it was mixed gently for 10 min. All the plates representing subgenomic library clones of Swarnaprabha were numbered and stored at −80 °C for long-term storage and use.

## 3. Results

### 3.1. Plant Growth

**Figure 3:**
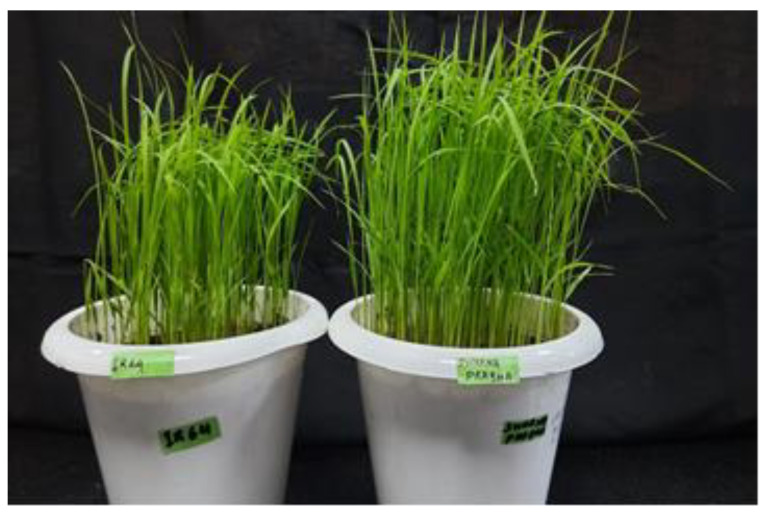
Morphologies ofIR-64 and Swarnaprabha 21-day-old seedlings grown in light-controlled growth chambers.

**Figure 4:**
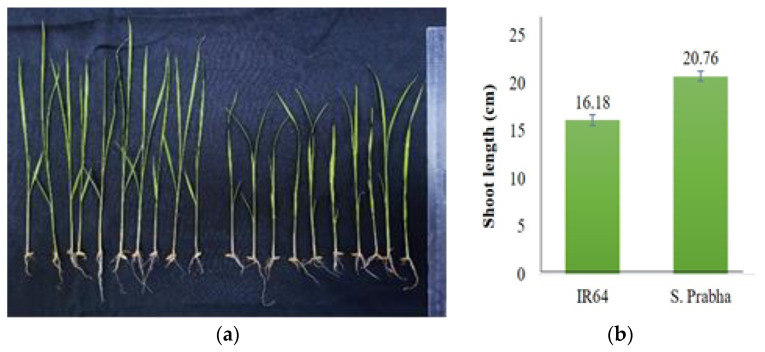
Growth parameters of contrasting rice cultivars. (**a**) 10 individual plants each of Swarnaprabha and IR-64, in response to low-light. (**b**) The average height of the plants of Swarnaprabha (20.76 cm) was found to be higher in comparison with that of IR-64 (16.18 cm).

Thirty seeds each of the *Oryza sativa* ssp. *indica* cultivars IR-64 and Swarnaprabha were germinated on filter paper. A germination rate of more than 80% was achieved for both rice cultivars. Morphologically, it was observed that IR-64 plants were shorter in height compared with those of Swarnaprabha cultivar (Figure 3). When the 21-day-old individual plants of both cultivars were compared, the plants of Swarnaprabha cultivar were found to be taller (20.76 cm) in comparison with those of IR-64 cultivar (16.18 cm) (Figure 4). Swarnaprabha cultivar is one of the low-light tolerant cultivars of rice, and it was identified in a field-level screening from the eastern rice-growing regions of India.

### 3.2. Isolation of Genomic DNA from Rice Tissue

**Figure 5:**
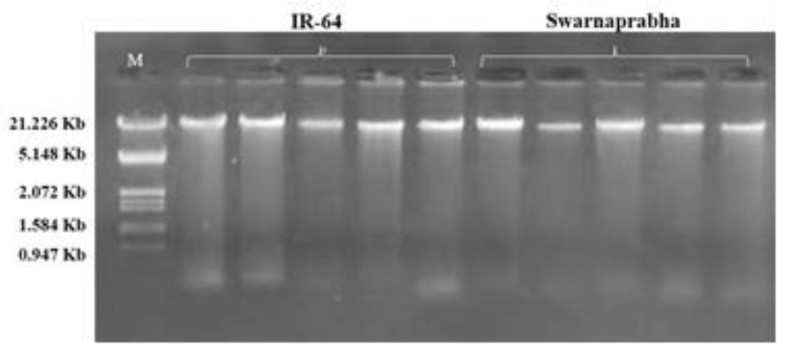
Isolation of genomic DNA from contrasting rice cultivars (IR-64 and Swarnaprabha). Lane M: Lambda DNA/*Eco*RI + *Hind*III ladder. The concentration of isolated genomic DNA in both samples, IR-64 (5 lanes) and Swarnaprabha (5 lanes), was found to be in a range of 500–800 ng/μL.

Young stem and leaf tissues of rice were harvested, and 0.1 gm of tissue was used to isolate the genomic DNA using the CTAB method. The quality and quantity of the isolated genomic DNA were good, as evident from the agarose gel electrophoresis (Figure 5). Because the isolation of good-quality DNA is a prerequisite for all down-stream molecular experiments, both agarose-gel-based observation and spectrophotometric readings were noted. The concentrations of genomic DNA isolated from IR-64 and Swarnaprabha were found to be in a range of 500–800 ng/μL. The spectrophotometric absorbance ratio at A_260/280_ was 1.87, while at A_260/230_, it was 2.15. These two ratios of isolated genomic DNA were ascertained to be of good quality and free of salt contamination.

### 3.3. Subgenomic Library Construction

**Figure 6:**
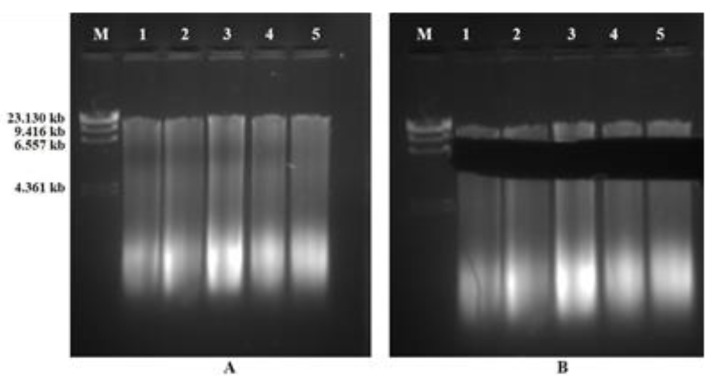
Partial restriction profile of Swarnaprabha genomic DNA. (**A**) Standardization of restriction of genomic DNA with *Eco*RI was carried out for 20′ (Lane 1), 40′ (Lane 2), 60′ (Lane 3), 90′ (Lane 4), and 120′ (Lane 5) durations. (**B**) Excised genomic size of 4–9 kb for ligation into pBluescript vector.

The isolated total genomic DNA of Swarnaprabha was used to prepare the subgenomic library for the identification/isolation of the photoreceptor genes from the low-light tolerant cultivar. The total genomic DNA was partially fragmented by restriction digestion with the *Eco*RI restriction endonuclease, and fragments ranging from 4 to 9 kb (Figure 6) were excised from the agarose gel and ligated to linearized pBluescript vector.

**Figure 7:**
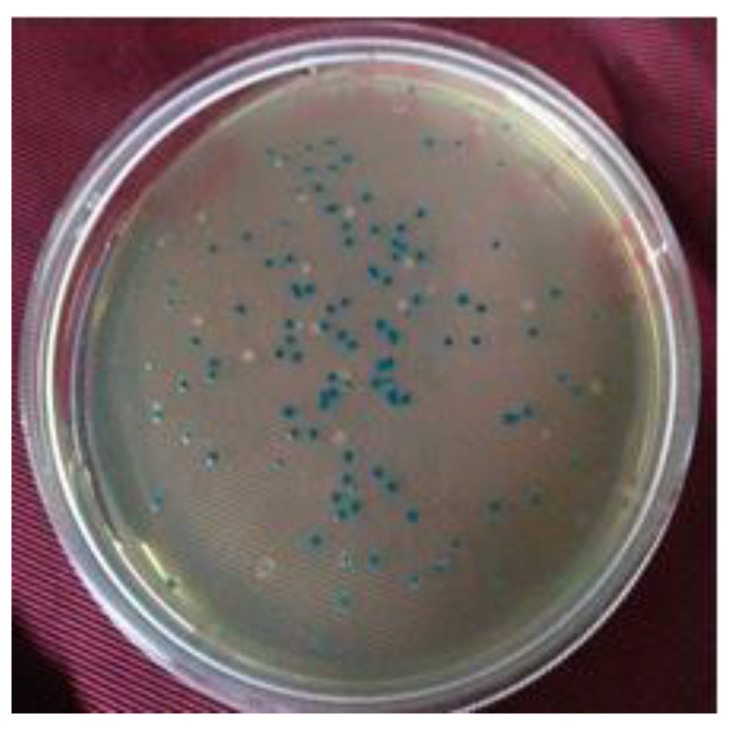
Blue–white screening of recombinant clones representing subgenomic library of Swarnaprabha by α-complementation. White colonies are recombinant clones containing subgenomic DNA of rice. Blue colonies are non-recombinant vector molecules (self-ligated pBluescript DNA).

The standardization of the restriction of genomic DNA was carried out for 0 min (control), 20′, 40′, 60′, 90′, and 120′ durations. It was found that the 30′ and 40′ *Eco*RI-restriction digestion of Swarnaprabha genomic DNA provided a smear from 1 kb to >10 kb. Thus, 40′ of *Eco*RI-restriction digestion (5 U/uL) was finally used for the genomic DNA restriction, and fragments that corresponded to 4–9 kb were eluted from the agarose gel. After ligation and transformation with competent cells, blue and white colonies were obtained on LA+ carbenicillin (100 μg/mL) containing X-Gal + IPTG (Figure 7).

**Figure 8:**
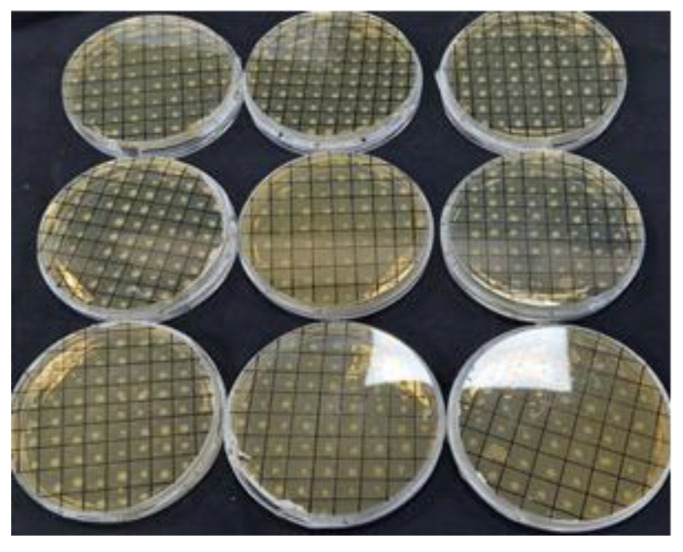
Axenic culture of subgenomic library of rice. Putative recombinant clones (white colonies) were restreaked on LA+ carbenicillin (100 μg/mL) grid plates supplemented with X-Gal +IPTG for α-complementation. *Bonafide* white colonies (representing recombinant clones) after two rounds of sub-culturing were stored individually for long-term storage.

The titer of the library was calculated as per the following formula:

N = ln(1 − P)/ln(1 − 1/F)

where N is the number of colonies, P is the probability (0.95), and F is the genome size/fragment size. Based on the above formula, the titer of our library was found to be 3.22 × 10^5^ cfu/mL. The white colonies from all the plates were picked and purified twice by streaking on LA+ carbenicillin (100 μg/mL) and X-Gal + IPTG plates (Figure 8).

### 3.4. Authentication of Library by Colony PCR

**Figure 9:**
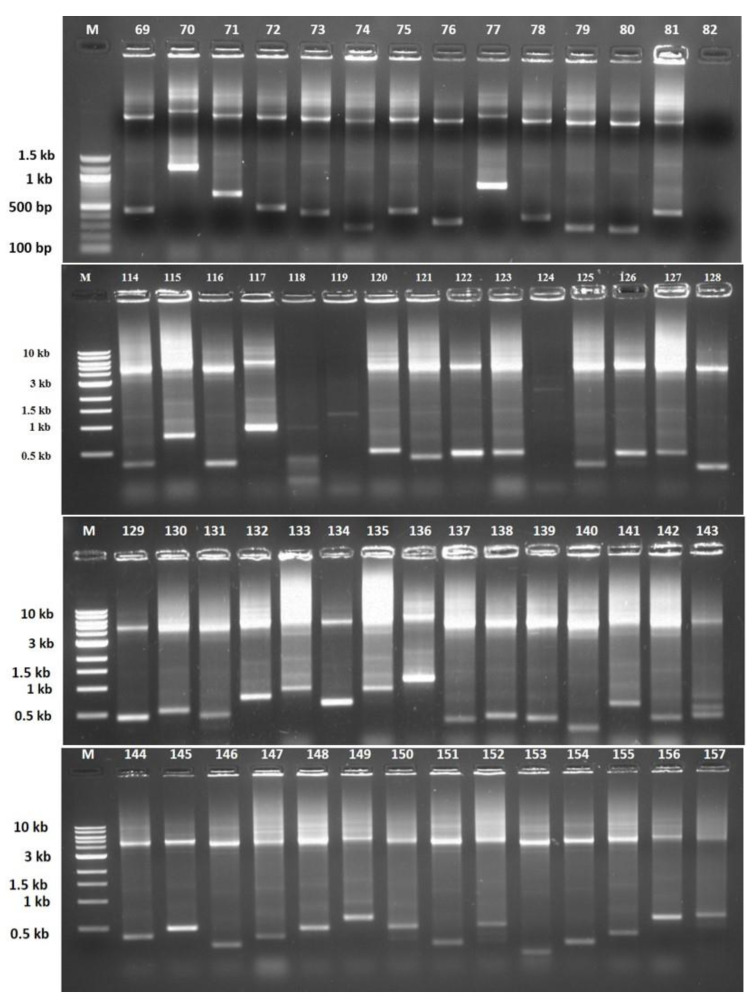
Colony PCR of randomly picked white colonies with universal M13 forward and reverse primers. Out of several colonies screened, sixty-six positive colonies were found to harbor different insert sizes. Lane M is the marker DNA of the indicated size, and Lanes 69–157 are the sixty-six positive clones.

The white colonies were restreaked in the form of a grid on LA+ carbenicillin plates for further screening (Figure 8). A number of putative positive white colonies were screened by colony PCR with vector-specific universal M13 forward and reverse primers flanking the multiple cloning site. For screening of the clones with colony PCR, the PCR conditions (extension time) were tuned to 30 s only to screen the clones for the presence or absence of foreign DNA. Sixty-six colonies were found to be positive for the presence of foreign DNA (insert) and were found to harbor different insert sizes (Figure 9).

**Figure 10:**
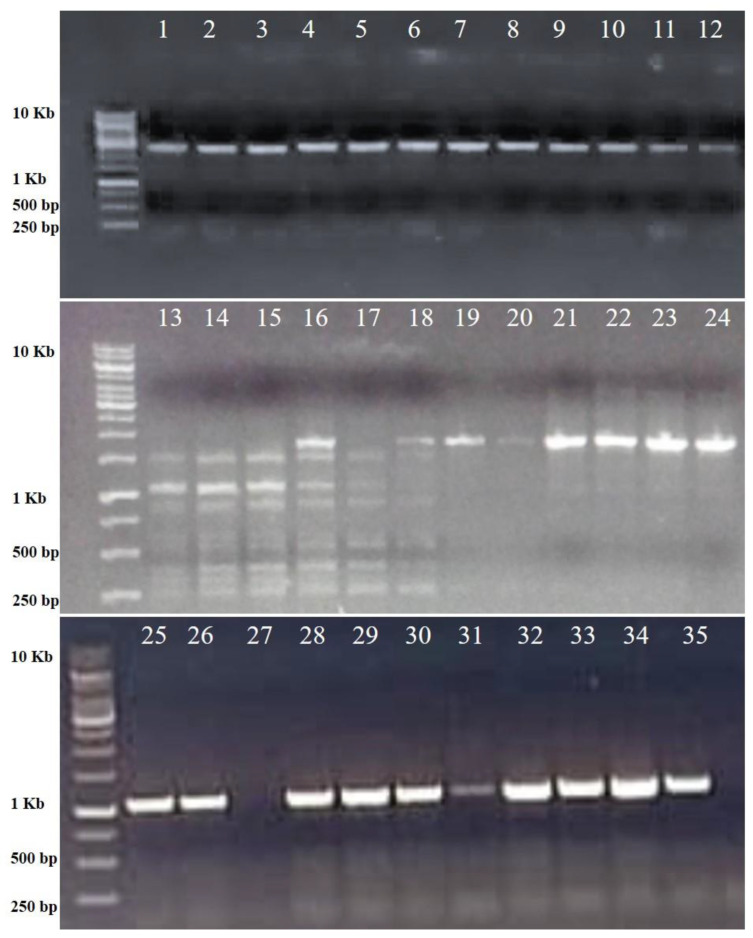
Colony PCR of randomly picked white colonies with gene-specific *phyA* forward and reverse primers. Out of 200 colonies screened, 35 positive colonies were found to harbor *phyA* genes (similar sizes but varied intensities). Lane M is the marker DNA of the indicated size, and Lanes 1–35 are thirty-five positive clones.

*PhyA* (*phytochrome A*) mediates irreversible photo-responses in the very-low-fluence and high-fluence ranges (VLFR and HFR), and primarily in the far-red (FR) spectral region [16]. For further authentication of the constructed subgenomic library, a set of 200 clones were screened for the presence of the *phyA* gene using gene-specific forward and reverse primers. For the screening of the clones with colony PCR, the PCR conditions (extension time) were tuned to one minute to screen the clones for the presence or absence of *phyA* genes. Two hundred colonies were screened for the presence of the *phyA*-specific gene. Out of two hundred randomly picked colonies, thirty-five clones yielded positive amplifications with varying intensities (Figure 10). The result reveals that the constructed genomic library of Swarnaprabha harbors, inter alia, photo-responsive genes.

### 3.5. Long-Term Storage of Subgenomic Clones

The axenic culture of recombinant clones that represent the subgenomic library of Swarnaprabha was repeatedly purified on LA+ carbenicillin (100 μg/mL) plates. A total of 3600 clones representing the subgenomic library were stored in 40% glycerol at −80 °C for future use.

## 4. Discussion

Photosynthetic plants are sessile organisms that have evolved to perceive and respond to the presence, quantity, and quality of light. They behave in an adaptive manner in response to changes in the ambient light intensity that prevail in their surroundings, either by being tolerant to shade and/or by avoiding shade. Plants assess the changes in light intensity by deploying phytochromes as photoreceptors, and they initiate a morphological response that is termed the shade-avoidance response (SAR) [34,35]. The SAR includes the elongation of the stem, hyponasty (i.e., the upward bending of the leaves), reduced tillering/branching, and early flowering. In field conditions, the SAR is the main parameter for setting the maximum planting densities for many crop plants [36,37]. Bright sunlight offers an equal amount of 600–700 nm red (R) and 600–700 nm far-red (FR) light to plants. While far-red light is largely reflected or transmitted, red light is absorbed by the green tissues. Canonically, the R/RF ratio is an indicator of the plant proximity and a cardinal parameter for shade avoidance. To date, the molecular mechanism for the SAR has been studied in *Arabidopsis* [38], but the detailed mechanisms of the signaling cascades and effectors for the regulation of plant growth under suboptimal light environments in rice are limited.

The predominant light receptors, phytochromes, exist in two photoconvertible isoforms: (i) the red-light-absorbing form (Pr) and (ii) the far-red light-absorbing form (Pfr). The Pr form is an inactive form that is present in the dark, which, after being triggered with R light, is converted to the active Pfr form. The Pfr form can again absorb FR light and be converted back to the Pr form. The active Pfr form is then translocated to the nucleus, giving phytochrome-related responses [18,39,40,41].

In the majority of plant species, the phytochromes are encoded by a small gene family. In *Arabidopsis*, there are five genes that encode phytochromes (*phyA*–*phyE*). Each phytochrome has a distinct physiological role that ensures the growth and development of the plant. However, *phyA* and *phyB* are the predominant phytochromes in the perception of light from ambience. *phyA* and *phyB* act antagonistically towards each other. For example, *phyB* plays a predominant role in the shade-avoidance response [38,42] that invigorates stem growth in low R/FR light, while it activates *phyA*, which inhibits stem elongation [43]. Phytochromes are synthesized in the dark in the inactive Pr form. Upon light absorption, the Pr form is reversibly converted to its active Pfr form, the activated Pfr form absorbs far-red light, and it is converted back to the Pr form. The biologically active Pfr form moves from the cytosol to the nucleus [18], and it regulates the down-stream gene expression as an activated transcription factor. Once inside the nucleus, Pfr interacts with the phytochrome-interacting factors (PIFs) [44,45], which are down-stream transcription factors [46], and which initiate photomorphogenesis-dependent plant growth and development [47,48,49].

To accomplish the objective of the construction of a subgenomic library of the low-light tolerant cultivar “Swarnaprabha”, the experimental plants were grown in shade and the control plants were grown in normal light. The 25% cut-off of natural light was chosen to mimic the low-light condition (experimental), while the replication of the same variety with a 0% cutoff was treated as natural light (control). Morphological changes in the contrasting genotypes (tolerant and susceptible), and notably the heights of the plants, were noted. Swarnaprabha, which has been identified as a low-light tolerant cultivar, recorded a higher length (20.76 cm) in comparison with the IR-64 cultivar (16.18 cm), which is identified as a low-light susceptible cultivar. Increasing their heights is one of the strategies adopted by plants to enhance their access to sunlight. Even in nature, plants mostly face competition for light from other plants of a similar height [50,51]. On the basis of the differences in the morphological features, such as the plant height and other agronomic traits, the Swarnaprabha cultivar was used for the construction of the subgenomic library for the isolation of photoreceptors.

Many advances have been made in the field of genomics [52,53,54] and transgenics [55,56] to impart biotic/abiotic tolerance [57,58] or engineer a trait in crops [59], but the genomic resources for low-light tolerance in plants are meagre. Therefore, a subgenomic library yielding a large number of clones that harbor photoreceptors or other agronomically important (intrinsic yield genes) traits is required. For the subgenomic library construction, genomic DNA was isolated from the shoots of Swarnaprabha, and the isolated DNA was found to be of good quality, free of salt, and amenable for down-stream applications, as evident from the gel electrophoresis and spectrophotometric studies. Further, the isolated genomic DNA was partially digested with *Eco*RI for the selection of the desired fragment size of 4–9 kb. This fragment size range was used for the construction of the subgenomic library because, as per the Gramene database, the *phyA* gene is from ~7 kb to ~9 kb in size (https://ensembl.gramene.org/Oryza_sativa/Gene/Summary?g=Os03g0719800;r=3:29168197-29176006;t=Os03t0719800-01, accessed on 10 March 2022). Hence, the subgenomic library was constructed using the genomic fragments of only this range to reduce the number of nonspecific clones. This helped to save time and resources during the screening of positive clones, and it increased the probability of identifying the *phyA* gene.

Subsequently, genomic DNA ranging in size from 4–9 kb was ligated into the pBluescript vector to aid the blue–white screening. The ligation product was used to transform the competent DH-5α strain of *E. coli*. Different dilutions of the transformation mix (1:10, 1:100, and 1:1000) were plated to test the correct dilution for plating to obtain well-separated colonies that could be further screened. It was found that a 1:100 dilution was the optimum dilution for plating. The titer of the library as per the given formula was estimated to be 3.22 × 10^5^ cfu/mL.

The transformed colonies were picked and streaked on LA + carbenicillin X-GAL + IPTG plates in the grid formation for screening. Out of several randomly picked colonies, sixty-six colonies were found positive by colony PCR for the presence or absence of genomic DNA. Vector-specific universal M13 forward and reverse primers were used for screening the recombinant clones, as both primer sequences are present in the pBluescript vector. In the PCR, the cycling conditions were set with an extension time of 30 s to facilitate an amplification of ~500–1000 bp, and this helped us to quickly screen a large number of colonies. Almost all the clones screened for the presence of insert DNA showed amplifications of varying sizes, confirming the insertion of genomic fragments and the good efficiency of our constructed library. The collection of clones representing the subgenomic library of Swarnaprabha was stored at −80 °C in glycerol for long-term storage for future usage.

To reconfirm, the generated library is enriched with photoreceptor genes, and another set of 200 random colonies were picked and screened for the presence of genes induced by low-light intensity, such as *phytochromeA* (*phyA*). The PCR reaction was set to obtain an amplification of varying intensity, and thirty-five clones with similar amplification sizes were picked. Our results confirm that the generated library is enriched in genes that are involved in photomorphogenesis, as the PCR targeted specifically for the light-induced *phyA* gene was positive. However, this does not preclude other clones from the recombinant pool of clones, as they might contain other intrinsic genes of interest from rice. Nevertheless, it did reconfirm the authenticity of the constructed subgenomic library as an efficient genomic resource representing a collection of recombinant clones (with a high titer) that can be used and shared among rice researchers for the isolation of any gene of interest from rice.

## 5. Conclusions

In conclusion, our results revealed the prevalence of variation amongst the rice genotypes in response to low-light conditions and the concurrent morphological differences to the induction of low-light. Based on the findings, the tolerant cultivar was used to construct a subgenomic library with a high titer. The clones generated from the subgenomic library of Swarnaprabha are an important genomic resource, and they can be utilized in the future for the isolation of phytochrome photoreceptors for fine-tuning the shade-avoidance response (SAR) in rice. It is important to determine the molecular basis of the variation in the yield in response to low-light among the photosensitive cultivars of Indian rice. In this regard, determining the allelic polymorphism in Swarnaprabha in comparison to the globally available rice genomic sequences in the future will be a fundamental discovery and will help in the development of low-light tolerant rice varieties.

## Figures and Tables

**Table 1:** *In vitro* conditions and constituents for cloning: restriction digestion (*Eco*RI) of pBluescript, and ligation of vector DNA with subgenomic rice fragments and PCR components for colony PCR verification of recombinant clones.

Vector DNA Preparation (*Eco*RI Digestion)		Vector (pBS) and Subgenomic DNA (Rice) Ligation		PCR Components	
Vector DNA (pBS)	20 μL	Vector (pBS)	8 μL	Template DNA	colony
*Eco*RI buffer	3 μL	Insert (4–9 kb)	15 μL	Forward Primer (0.5 μM)	0.5 μL
*Eco*RI (Enzyme)	2 μL	Ligase buffer	3 μL	Reverse Primer (0.5 μM)	0.5 μL
Water	5 μL	T4 DNA ligase	2.5 μL	dNTPs (0.2 mM)	0.5 μL
Total	30 μL	Water	1.5 μL	PCR Buffer (10X)	2.0 μL
		Total	30 μL	Taq polymerase (2 units)	0.5 μL
				Water	16 μL
				Total	20 μL

**Table 2:** Sequences of primers used for colony PCR. Vector-specific universal M13 forward and reverse primer sequences.

S. No.	Primer Name	Primer Sequence
1	M13 Forward Primer	AGCGGATAACAATTTCACACAGG
2	M13 Reverse Primer	CCCAGTCACGACGTTGTAAAACG

**Table 3:** Sequence of gene-specific primers used for colony PCR.

S. No.	Primer Name	Primer Sequence
1	*phyA* Forward Primer	CATCCATCGAGTTCTAGCAGG
2	*phyA* Reverse Primer	AAATCAATCAACCCACAACG

## Data Availability

Not applicable.
